# Effects of oral moisturizing gel containing propolis following head and neck radiotherapy: randomized controlled pilot trial

**DOI:** 10.1038/s41405-021-00068-3

**Published:** 2021-02-25

**Authors:** Ryoma Nakao, Takao Ueno

**Affiliations:** 1 Department of Bacteriology I, National Institute of Infectious Diseases, Tokyo, Japan; 2 Department of Dentistry, National Cancer Center Hospital, Tokyo, Japan

**Keywords:** Oral microbiology, Mucositis

## Abstract

**Aim:**

Topical administration of oral gel may reduce radiotherapy-related oral complications. The aim of this study was to examine clinical and microbiological effects of self-administration of different gel formulations to oral mucosa in head and neck cancer patients.

**Materials and methods:**

Twenty-seven subjects were recruited from outpatients who underwent radiotherapy of at least 50 Gy to the head and neck area. They were randomly assigned to oral gel with the following different ingredients: placebo, chlorhexidine, curry leaf, propolis, and turmeric. Before and after intervention, oral symptoms were evaluated, and nine oral pathogens in saliva were also quantified using real-time PCR.

**Results:**

Twenty-five subjects completed the study and their data were analyzed. The number of *Porphyromonas gingivalis* in saliva significantly decreased after treatment with propolis gel, but not after any other treatments. Propolis gel treatment also relieved oral pain in all subjects who had oral pain at the baseline.

**Conclusions:**

Topical administration with propolis gel may not only reduce *P. gingivalis* carriage in saliva, but also relieve oral pain.

**Discussion:**

A future larger-scale clinical trial of oral propolis gel is needed to determine its clinical efficacy in radiotherapy-related oral complications of head and neck cancer patients.

## Introduction

Radiotherapy in the head and neck area is usually intense, high dose, and continuous, and often used in conjunction with chemotherapy, thus acute and late radiotherapy-related complications frequently occur.^1^ A previous report noted that radiation-induced oral mucositis was found in up to 100% of head and neck cancer patients who received a dosage of 25 Gy.^2^ Resultant damage to oral mucosa, mainly due to injury of epithelial and lamina propria cells and/or salivary gland cells, results in a wide range of the complications, including pain, dryness, ulceration, and pseudomembranous formation, as well as infectious diseases in the oral cavity.

We focused here on use of various oral moisturizing gel formulations with different natural products for head and neck cancer-affected individuals, and assessed the clinical effects as well as microbiological effects including antimicrobial activity. One of the natural products added was curry leaf (*Murraya koenigii*), frequently used for flavoring curries and chutneys, which has been shown to have antibacterial activities against a range of pathogens.^3,4^ Another product chosen for testing was turmeric, a polyphenolic compound isolated from *Curcuma longa*, because of its known in vitro antibacterial activity.^5,6^ In addition, we chose propolis, which is a complex of resinous compounds collected by bees and has been utilized for many years in folk medicine for many years.^7^ In our recent study, we have reported a mechanism of the antibacterial activity of propolis against the major periodontopathic bacterium *Porphyromonas gingivalis*.^8^ Propolis at the final concentration of 100 μg/mL inhibited both the growth and biofilm formation of *P. gingivalis*. In addition, *P. gingivalis* is relatively susceptible to propolis among oral bacterial species.^8^ Very recently, we have also reported that administration of a propolis ointment into periodontal pockets not only reduced the amount of *P. gingivalis* in gingival crevicular fluid, but also improved clinical attachment level (CAL),^9^ which is regarded as one of the most clinically relevant parameters for periodontitis.^10^

The present pilot study findings indicate that treatment with propolis gel may not only decrease the number of *P. gingivalis* organisms in saliva, but also relive oral pain. Clinical implications for use of an oral moisturizing gel, particularly that containing propolis, to reduce radiotherapy-related oral complications are also discussed.

## Materials and methods

### Study design

The protocol was designed in accordance with the Declaration of Helsinki as a statement of ethical principles, and received approval from the Clinical Research Ethics Committees of both National Cancer Center Hospital (No. 2016-081) and National Institute of Infectious Diseases (No. 680). This study has been registered in the University Hospital Medical Information Network in the Japan Clinical Trials Registry (No UMIN000023016). Patients who underwent radiotherapy with a total dosage of at least 50 Gy for cancer of the head and neck area were assessed for eligibility, and enrolled at the Department of Dentistry, National Cancer Center Hospital (Tokyo, Japan) between October 2016 and February 2017. Informed consent was obtained from each subject before the study. Details regarding patient selection for the trial are described in Supplementary Text S1.

**Fig. 1:**
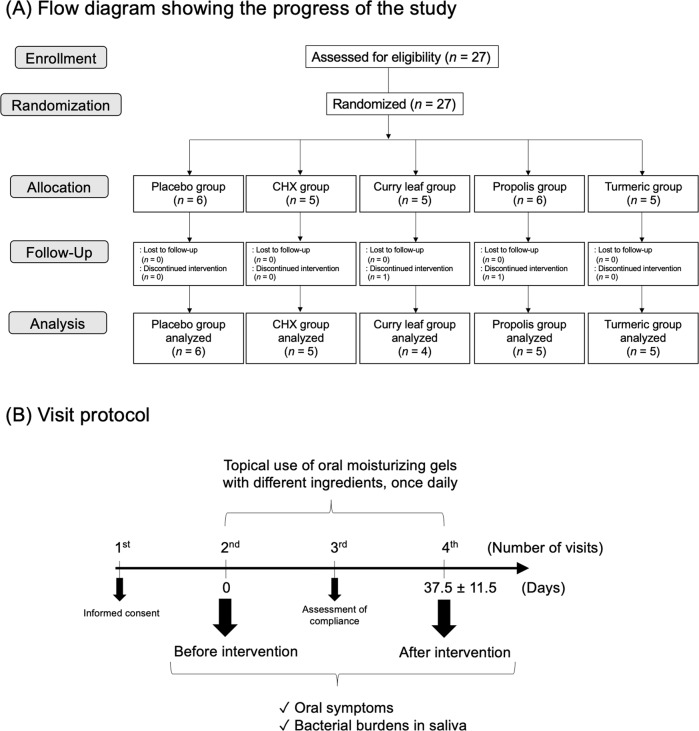
Overview of the present study. **A** Flow diagram of study protocol. Twenty-seven subjects who fulfilled the entrance criteria were registered between October 2016 and February 2017. They were randomly divided into five groups and given a specific oral moisturizing gel formulation to be applied at home. Each oral moisturizing gel contained one of the following five ingredients: placebo, chlorhexidine, curry leaf, propolis, or turmeric. All except two dropout patients, who were assigned to the curry leaf and propolis groups (i.e., 25 subjects), were followed and analyzed. **B** Visit protocol. At the first visit, informed consent was obtained from all patients. Sampling of saliva was performed before and after topical use of the oral moisturizing gels with different ingredients for 37.5 ± 11.5 days, i.e., at the second and fourth visits. To improve compliance with topical use of the gels at home, we checked compliance by interviewing each subject on an intermediate day of the intervention period, i.e., at the third visit. Of these participants, all except two patients who dropped out (25 subjects) were eventually enrolled and analyzed in the present study.

Twenty-seven subjects in total were enrolled and randomly divided into five groups, then subsequently received intervention treatment with an oral moisturizing gel containing one of the following five ingredients: placebo, chlorhexidine gluconate (CHX), curry leaf, propolis, and turmeric. Of the 27 participants, all except two dropout patients eventually underwent follow-up examinations and their results were analyzed (Fig. 1A, Table 1). Block randomization of all subjects was performed using a computer-generated random number table performed by staff members external to the study. Blinded, numbered gels were used by the patients assigned the same ID numbers. Informed consent was obtained at the time of the first visit, then sampling was performed before and after intervention, i.e., at second and fourth visits (Fig. 1B). The mean of intervention period was 37.5 ± 11.5 days (Fig. 1B). At third and fourth visits (Fig. 1B), we checked the records of individual administration, as well as interviewed the patients regarding whether oral administration of the gel was properly performed, their impression of the gel product after trying it, and occurrence of any adverse reactions.

**Table 1:** Characteristics of 25 subjects^a^ at baseline visit.

Characteristic	Descriptive statistic	Placebo group	CHX group	Curry leaf group	Propolis group	Turmeric group	All group
Number of subjects	*n*	6	5	4	5	5	25
Male	*n* (%)	4 (66.6)	1 (20.0)	3 (75.0)	3 (60.0)	5 (100.0)	16 (64.0)
Female	*n* (%)	2 (33.3)	4 (80.0)	1 (25.0)	2 (40.0)	0 (0.0)	9 (36.0)
Age	year [mean ± SD]	65.2 ± 9.3	57.2 ± 15.6	54.8 ± 23.0	63.0 ± 16.3	53.4 ± 15.5	59.1 ± 15.2
Age range	range [min–max]	55–77	43–83	21–67	39–78	28–55	21–83
Subjective evaluation							
Number of subjects with oral pain	*n* (%)	3 (50.0)	1 (20.0)	1 (25.0)	4 (80.0)	4 (80.0)	13 (52.0)
Oral mucosal pain (VAS score)	mm [mean ± SD]	17.0 ± 15.7	22.6	20.8	44.1 ± 31.0	37.3 ± 21.2	32.3 ± 23.0
	range [min–max]	5.6–34.9	22.6	20.8	9.4–76.4	13.2–59.4	0.0–76.4
Number of subjects with dryness feeling	*n* (%)	6 (100)	5 (100.0)	4 (100.0)	4 (80.0)	4 (80.0)	23 (92.0)
Oral dryness (VAS score)	mm [mean ± SD]	66.2 ± 39.3	49.2 ± 24.2	57.8 ± 38.5	67.5 ± 21.1	71.5 ± 19.2	60.3 ± 32.2
	range [min–max]	6.6–100.0	22.6–72.6	24.5–100.0	48.1–92.4	46.2–88.8	6.6–100.0
Objective evaluation							
Oral hygiene (visual inspection)	score [mean ± SD]	2.7 ± 0.5	2.8 ± 0.4	3.0 ± 0.0	2.8 ± 0.4	2.8 ± 0.4	2.8 ± 0.4
Candidiasis (visual inspection)	*n*	0	0	0	0	0	0
Radiotherapy							
Total dose of radiation (Gy)	Dose [mean ± SD]	67.3 ± 2.1	60.8 ± 7.3	69.0 ± 2.0	69.5 ± 2.0	60.8 ± 9.1	65.4 ± 6.3
	range [min–max]	66.0–70.0	52.0–70.0	66.0–70.0	66.0–71.4	50.0–70.0	50.0–71.4
Surgical therapy	*n* (%)	2 (33.3)	1 (20.0)	4 (100.0)	3 (60.0)	3 (60.0)	13 (52.0)
Chemotherapy	*n* (%)	4 (66.6)	4 (80.0)	1 (25.0)	4 (80.0)	2 (40.0)	15 (60.0)

^a^Shown are data from 25 subjects who completed the entire course of the planned intervention as well as sampling.

### Oral moisturizing gel with natural products

A commercially available oral moisturizing gel (Oral Aqua Gel^®^ flavored with raspberry, GC, Tokyo, Japan) was used as the base for preparing gels containing the three different natural products (curry leaf, propolis, or turmeric) or the antimicrobial biocide chlorhexidine. The main ingredients of the base gel were diglycerin as moisturizer, carboxymethylcellulose sodium and carrageenan as thickeners, sodium citrate as pH adjuster, and methylparaben as preservative. Detailed information regarding the natural products and biocide used in this study and preparation of each gel are described in Supplementary Text S1.

### Sample collection

At the first visit, a trained dental hygienist instructed the subject regarding how to collect saliva. On the morning the second and fourth visits, each subject collected stimulated whole saliva at home by themselves before brushing their teeth. Samples submitted were transported at 4 °C to the National Institute of Infectious Diseases laboratory. Procedures related to sample preparation for DNA isolation and real-time PCR analysis are described in Supplementary Text S1. We have chosen the following nine pathogens for the real-time PCR analysis: *P. gingivalis*, *Tannerella forsythia*, *Treponema denticola*, *Fusobacterium nucleatum*, *Aggregatibacter actinomycetemcomitans*, methicillin-resistant *Staphylococcus aureus* (MRSA), *Escherichia coli*, *Serratia marcescens*, and *Candida albicans*.

### Topical administration of oral gel

Regarding the technique for self-administration of the gel at home, a trained dental hygienist instructed the subjects, as follows: (1) use once at every night after brushing teeth for 1 month during the intervention period. (2) Administer 1 mL of gel to the whole oral mucosal surface using a disposable brush with a polyurethane sponge head (MHG250, Molten, Tokyo, Japan).

### Primary and secondary endpoints

The primary endpoint of this study was resolution of oral symptoms after a 1-month administration of the gels assigned to the different arms. The secondary endpoints were clearance of oral pathogens after the gel application in each arm. See Supplementary Text S1 for descriptions of “Evaluation of clinical parameters” and “Evaluation of microbiological parameters”.

### Statistical analysis

Statistical analysis was done with GraphPad Prism version 8 for Macintosh (GraphPad Software, San Diego, CA). One-way analysis of variance followed by Dunnett’s multiple comparison test was used to statistically evaluate clinical and microbiological variables in each group. Using the mean values of the respective measurements before and after treatment, changes over time were calculated and tested with a Mann–Whitney U test. All values are expressed as the mean ± SD. *P* values of 0.05 or less were considered to be statistically significant.

## Results

Due to two dropout patients, a total of 25 subjects completed the 1-month intervention trial (Fig. 1A). None (0%) had difficulties with application, while the viscosity of the gel was noted as unpleasant by one (4%) subject. One hundred percent of patients (25/25) had no difficulties in gel application. Characteristics of the 25 subjects (16 men and 9 women), between 21 and 83 years of age (mean 59.1 ± 15.2), are shown in Table 1. At the baseline visit, 92% of subjects felt dryness in the oral cavity, while 52% of subjects had pain in the oral cavity. No emergence of candidiasis was observed by visual inspection. At baseline, the total radiation doses of all subjects were 65.4 ± 6.3 Gy, with no statistical significance between the groups.

We examined the effect of each intervention on bacterial clearance in saliva by species-specific real-time PCR analysis. Microbiological assessment of saliva showed that the total number of oral bacteria was not influenced by topical application of gel, irrespective of the ingredients (Supplementary Fig. S2). Detection frequencies of each bacterium at baseline were different: high frequency for *F. nucleatum* (100%, 25/25) and *T. forsythia* (96%, 24/25); moderate frequency for *P. gingivalis* (60%, 15/25), *T. denticola* (60%, 15/25), and *C. albicans* (64%, 16/25); low frequency for *A. actinomycetemcomitans* (8%, 2/25) and MRSA (14.0%, 7/25). *S. marcescens and E. coli* were not detected in saliva of any subjects.

**Fig. 2:**
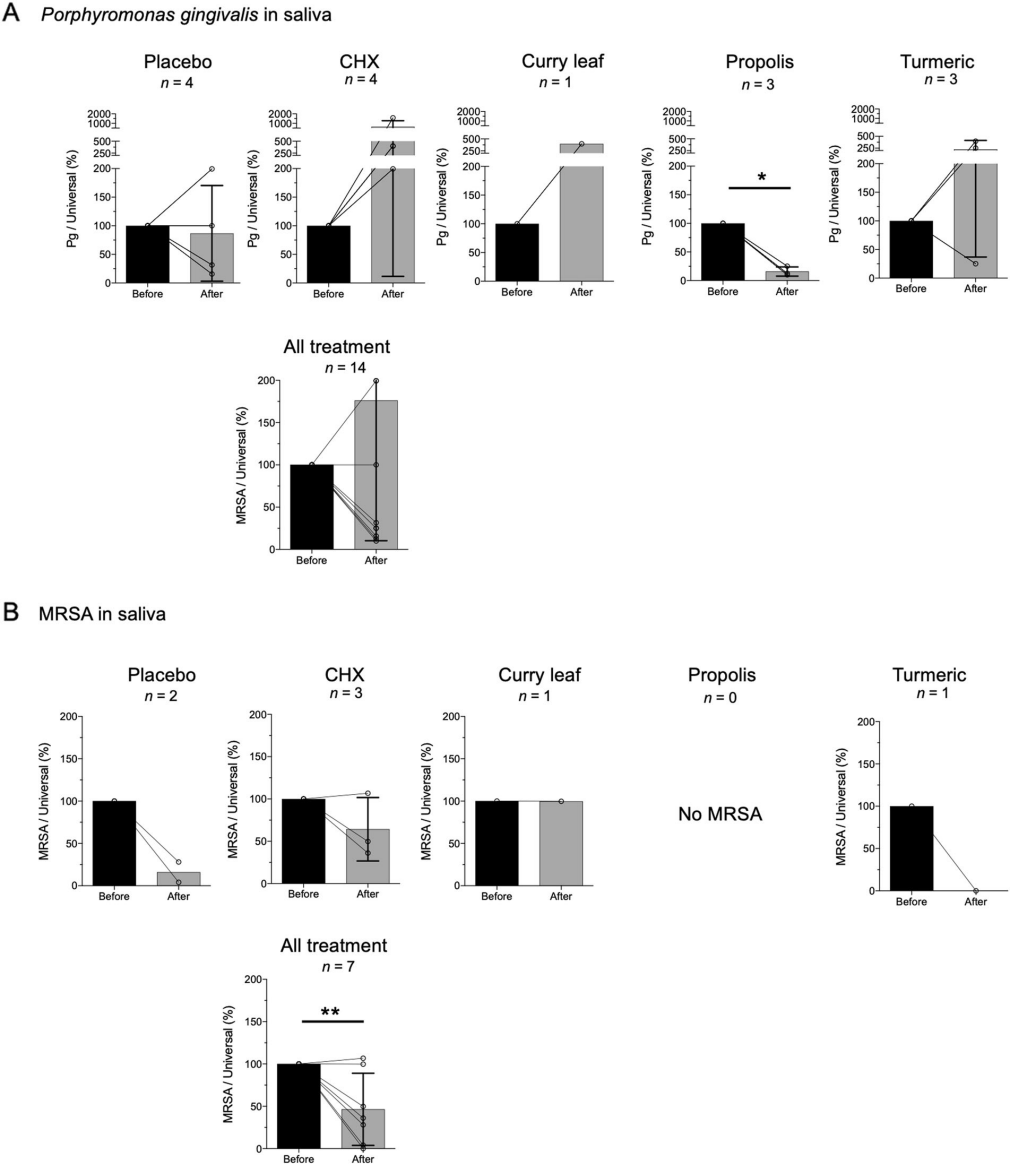
Effect of oral gels with different ingredients on bacteria in saliva. Transition of the numbers of **A** *P. gingivalis* and **B** MRSA before and after the intervention was assessed by quantitative real-time PCR. The copy number of each species-specific gene was normalized by that of the universal 16S-rRNA gene. The normalized values at baseline (before) and post-intervention (after) were standardized as 100% and the ratio (%) to the baseline value, respectively, then were plotted as open circles. The open circles before and after the intervention for each individual are connected with a line. The average with SD of “before” and “after” are indicated as black and gray bars with lines, respectively. *P ≤ 0.05; **P ≤ 0.01.

It is interesting to note that the number of *P. gingivalis* organisms was decreased in the propolis gel treatment group (*P* ≤ 0.05, Fig. 2A), but not in the others (data not shown). Moreover, despite the low detection frequency of MRSA, when data from all subjects were analyzed together, MRSA carriage in saliva was shown to be reduced (*P* ≤ 0.01, Fig. 2B).

**Fig. 3:**
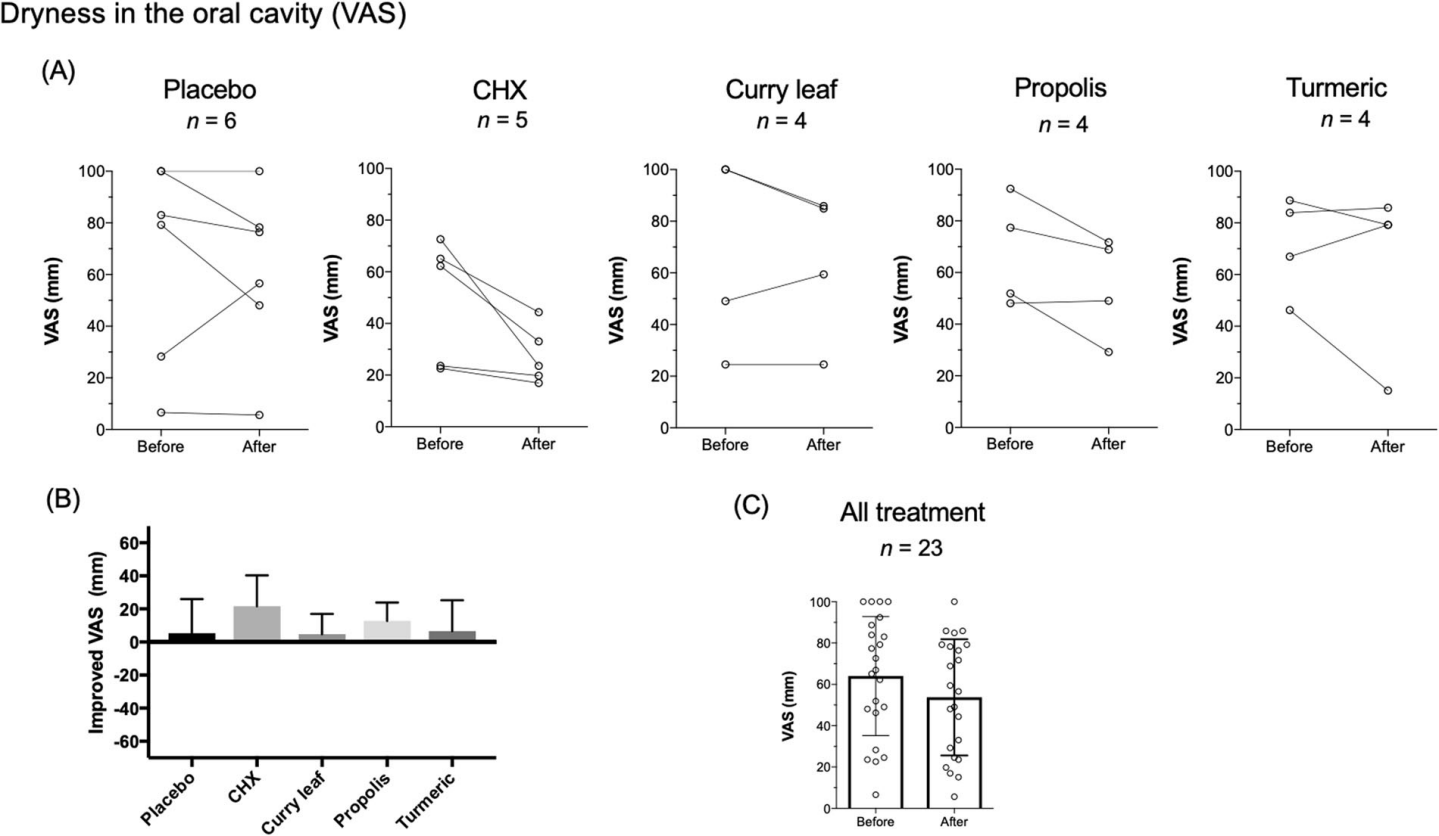
Effect of intervention on dryness in the oral cavity. **A** Evaluation of dryness in the oral cavity in each group. The transition of the VAS of dryness (mm) in every patient before and after the intervention is denoted by circles and the connecting line. **B** Improved VAS of dryness (mm) between every group. **C** Evaluation of dryness in the oral cavity in all treatment group. The bar graph of VAS of dryness (mm) with average ± SD is shown. VAS scores of individual subjects (*n* = 23) are also plotted in the graph.

**Fig. 4:**
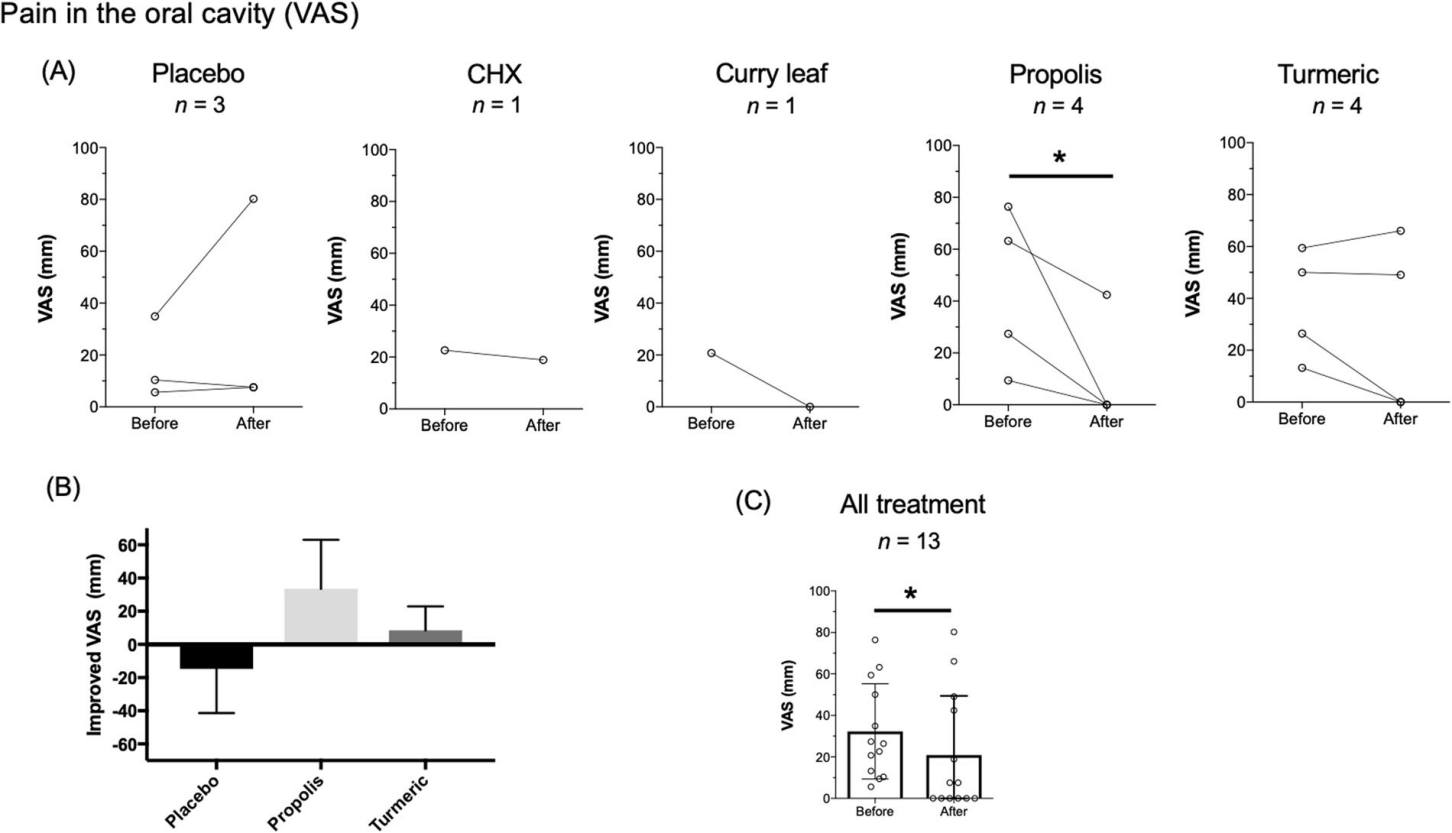
Effect of intervention on oral mucosal pain. **A** Evaluation of mucosal pain in the oral cavity in each group. The transition of the VAS of oral mucosal pain (mm) in every patient before and after the intervention is denoted by circles and the connecting line. **B** Improved VAS of oral mucosal pain (mm) between every group. **C** Evaluation of oral mucosal pain in all treatment groups. The bar graph of VAS of oral mucosal pain (mm) with average ± SD is shown. VAS scores of individual subjects (*n* = 13) are also plotted in the graph. *P ≤ 0.05.

The effect of the intervention on dryness (Fig. 3) and mucosal pain (Fig. 4) in the oral cavity was also examined. At baseline, most patients felt dryness in the oral cavity (Table 1). Of the five groups, all subjects in CHX and propolis groups tended to improve dryness in the oral cavity, but with no statistically significant difference between before and after intervention (Fig. 3A). All five groups tended to improve the VAS scores of oral dryness after the intervention (Fig. 3B). When all subjects were analyzed, the oral moisturizing gel tended to improve dryness in the oral cavity, but with no statistically significant difference (Fig. 3C). Regarding oral mucosal pain, at baseline, there were approximately half of patients who felt oral mucosal pain (Table 1). Oral pain was significantly relieved by propolis gel treatment (*P* ≤ 0.05), but not by the other treatments (Fig. 4A). The level of improvement in the propolis group was higher than that of the placebo and the turmeric group, but the difference between the three groups was not statistically significant (Fig. 4B). When all subjects were analyzed, the use of oral moisturizing gel was found to relieve pain in the oral cavity at a statistically significant level (*P* ≤ 0.05, Fig. 4C). As for oral hygiene, gingivitis, and oral moisture measured by Mucus^®^, we did not observe any improvement in each group, and there was no statistically significant difference between the groups (Supplementary Fig. S1).

## Discussion

Several reports have demonstrated the clinical usefulness of oral moisturizing gel for head and neck cancer patients who received different types of radiation treatments.^11–13^ However, there is no gold standard protocol for prevention or reduction of oral dryness and pain in post-radiotherapy patients, and the benefits of oral moisturizing gel use is controversial.^14–17^ In this study, moisturizing gels containing different ingredients were self-administrated to oral mucosa using a disposable brush with a soft sponge head once daily for approximately 1 month, following careful application instructions from a trained dental hygienist. When the results of the total cohort were analyzed, oral mucosal pain was significantly improved after the 1-month intervention (Fig. 4). Notably, of the 13 patients who had a pain at baseline, 6 patients did not feel pain in the oral cavity at all after completion of the 1-month study (Fig. 4). Unfortunately, the placebo effect, as well as the possibility of self-healing occurring during the intervention period, was not considered in the present clinical study. Nevertheless, previously we have reported that oral pain was not relieved by a moisturizing micro-gel spray application for oral complications in 18 patients who received head and neck radiotherapy and/or chemotherapy, when the spray was used as required for only 1 week.^13^ So, the regimen used in the present study appears to be superior to that of the micro-gel spray intervention, in terms of the higher frequency (once daily) and longer duration (1 month) of oral gel use.

Our results also expand current knowledge regarding the effects of topical gel application on a range of periodontal and opportunistic pathogens in the oral cavity. Notably, when results from the full cohort were analyzed together, the burden of MRSA in saliva was significantly reduced (Fig. 2B). Although the number of subjects is too few to conclude regarding the microbiological efficacy of the oral moisturizing gel, to the best of our knowledge this is the first study of the effectiveness of topical gel administration for clearance of MRSA from the oral cavity. Further investigation in a larger clinical trial will allow a more definitive conclusion regarding the benefit of the self-administration of oral gel.

In the present study, we have found that patient burden related to *P. gingivalis* was significantly decreased after treatment with the propolis gel (Fig. 2A). In addition, oral pain was significantly relieved by propolis gel treatment (Fig. 4). Recently, we have reported that propolis showed a rapid bactericidal effect on *P. gingivalis* in vitro study.^8^ More recently, we have reported that administration of propolis ointment into periodontal pockets eliminated *P. gingivalis* in gingival crevicular fluid and improved CAL in a clinical trial of patients with periodontitis.^9^ The findings in the present study are consistent with those of previous research on antimicrobial activity against *P. gingivalis*. On the other hand, propolis diminishes inflammation and facilitates wound healing.^18–20^ In this regard, we speculate that oral pain was significantly relieved by propolis gel treatment (Fig. 4A) thanks to anti-inflammatory and wound-healing activities of propolis.^18–20^ Taken together, we suggest that propolis may be a beneficial adjunct to not only reduce *P. gingivalis* burdens in the oral cavity, but also relieve oral mucositis in patients who received head and neck radiotherapy.

When designing a pilot trial, sample size is a critical consideration. Whitehead et al. recommended pilot trial sample sizes per treatment arm of 75, 25, 15, and 10 for standardized effect sizes that are extra small [≤0.1], small [0.2], medium [0.5], or large [0.8], respectively.^21^ As compared with those recommended values, the sample size of our pilot study is relatively small. Therefore, we recognize the possible risk of false positive results, and/or over-estimation of the association between intervention and outcome in this study. Nevertheless, after careful interpretation, the present data should be considered useful for designing a future larger-scale clinical trial with a longer follow-up period, which could provide additional evidence of the effectiveness of such a preparation.

## Supplementary information

Supplementary Information
