# Supplementary material for: Effects of oral moisturizing gel containing propolis following head and neck radiotherapy: randomized controlled pilot trial
Source: BDJ Open. 2021 Feb 25;7:12. doi: 10.1038/s41405-021-00068-3 (PMC7907078; doi:10.1038/s41405-021-00068-3)
Supplement: Supplementary file 1 — Supplementary Information [file 41405_2021_68_MOESM1_ESM.pdf]

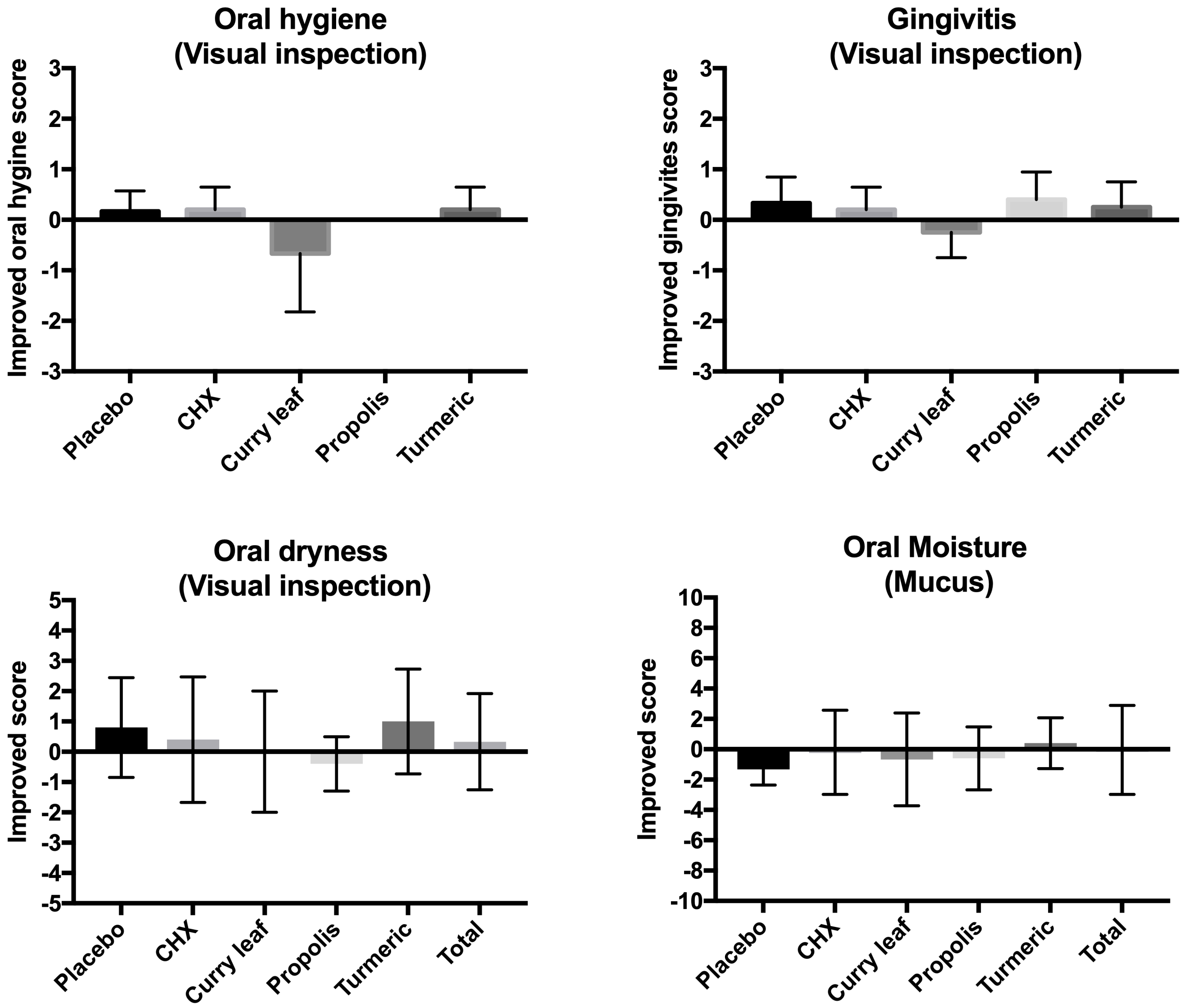

Fig. S1. Nakao et al.

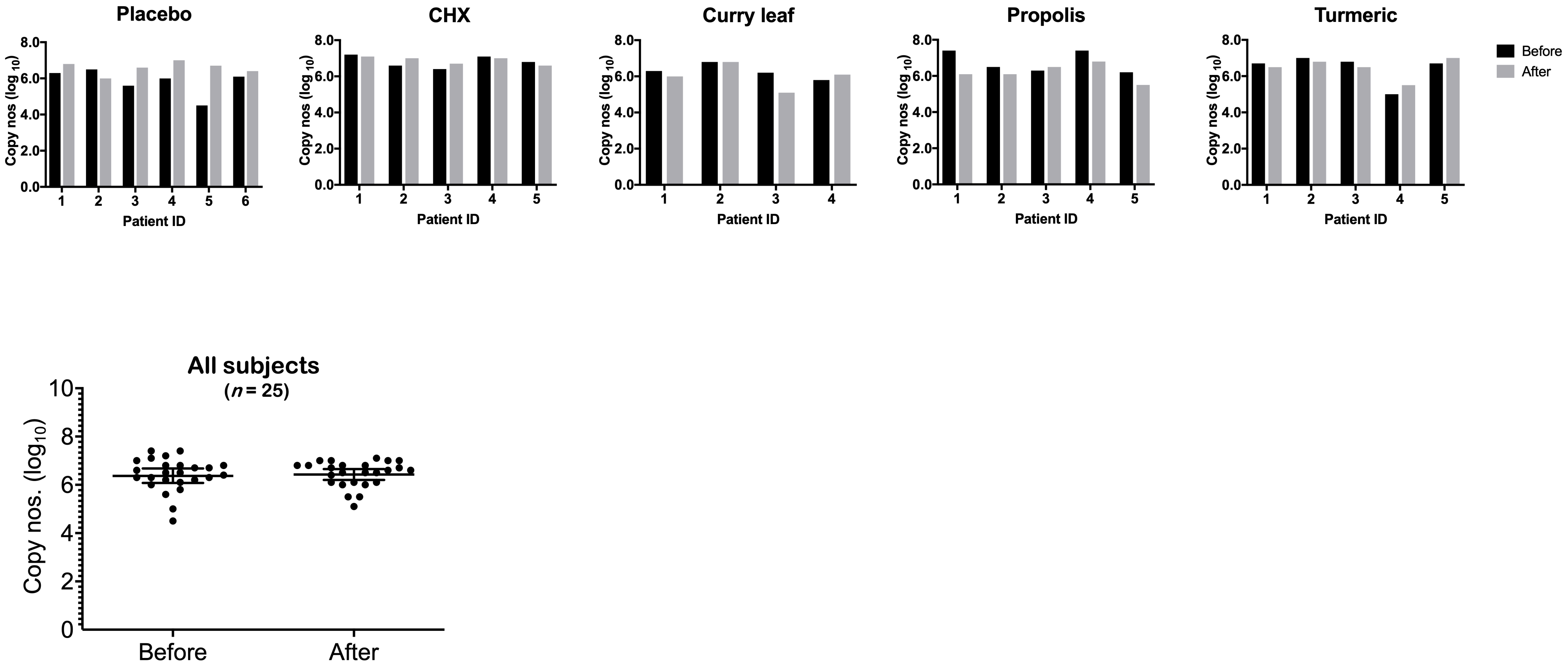

Fig. S2. Nakao et al.

Table S1. Primers and fluorescent probes used in this study

Names	Bacteria	Target	Sequence (5' to 3')	Reference
Universal-F	Pan-bacteria	16S rRNA	TCCTACGGGAGGCAGCAGT	(Nadkarni et al. 2002)
Universal-R	Pan-bacteria	16S rRNA	GGACTACCAGGGTATCTAATCCTGTT	(Nadkarni et al. 2002)
Universal-probe	Pan-bacteria	16S rRNA	(6FAM)-CGTATTACCGCGGCTGCTGGCAC-(TAMRA)	(Nadkarni et al. 2002)
Pg1198-F	*P. gingivalis*	16S rRNA	TACCCATCGTCGCCTTGGT	(Yoshida et al. 2003)
Pg1323-R	*P. gingivalis*	16S rRNA	CGGACTAAAACCGCATACACTTG	(Yoshida et al. 2003)
Pg1238T	*P. gingivalis*	16S rRNA	(6FAM)-GCTAATGGGACGCATGCCTATCTTACAGCT-(TAMRA)	(Yoshida et al. 2003)
Tf-F	*T. forsythia*	16S rRNA	TGAAAGTTTGTCGCTTAACGATAAAA	(Tada et al. 2012)
Tf-R	*T. forsythia*	16S rRNA	TCGTGCTTCAGTGTCAGTTATACCT	(Tada et al. 2012)
Tf-Probe	*T. forsythia*	16S rRNA	(6FAM)-AGATGAAGTAGGCGGAATGCGTGGTGTA-(TAMRA)	(Tada et al. 2012)
Td-F	*T. denticola*	16S rRNA	CCGAATGTGCTCATTTACATAAAGGT	(Kuboniwa et al. 2004)
Td-R	*T. denticola*	16S rRNA	GATACCCATCGTTGCCTTGGT	(Kuboniwa et al. 2004)
Td-probe	*T. denticola*	16S rRNA	(6FAM)-ATGGGCCCGCGTCCCATTAGC-(TAMRA)	(Kuboniwa et al. 2004)
Aa1956-F	*A. actinomycetemcomitans*	*hlyA*	CAGCATCTGCGATCCCTGTA	(Yoshida et al. 2003)
Aa2102-R	*A. actinomycetemcomitans*	*hlyA*	TCAGCCCTTTGTCTTTCCTAGGT	(Yoshida et al. 2003)
Aa2034T	*A. actinomycetemcomitans*	*hlyA*	(6FAM)-TCGAGTATTCCTCAAGCATTCTCGCACG-(TAMRA)	(Yoshida et al. 2003)
Fn619F	*F. nucleatum*	23S rRNA	CGCAGAAGGTGAAAGTCCTGTAT	(Yoshida et al. 2003)
Fn719R	*F. nucleatum*	23S rRNA	TGGTCCTCACTGATTCACACAGA	(Yoshida et al. 2003)
Fn663T	*F. nucleatum*	23S rRNA	(6FAM)-ACTTTGCTCCCAAGTAACATGGAACACGAG-(TAMRA)	(Yoshida et al. 2003)
Pi-F	*P. intermedia*	16S rRNA	TCCACCGATGAATCTTTGGTC	(Kuboniwa et al. 2004)
Pi-R	*P. intermedia*	16S rRNA	ATCCAACCTTCCCTCCACTC	(Kuboniwa et al. 2004)
Pi-probe	*P. intermedia*	16S rRNA	(6FAM)-CGTCAGATGCCATATGCGGACAACATCG-(TAMRA)	(Kuboniwa et al. 2004)
MRSA-F	MRSA	*mecA*	AAAGAACCTCTGCTCAACAAGT	(Kuboniwa et al. 2004)
MRSA-R	MRSA	*mecA*	TGTTATTTAACCCAATCATTGCTGTT	(Kuboniwa et al. 2004)
MRSA-probe	MRSA	*mecA*	(6FAM)-CCAGATTACAACTTCACCAGGTTCAACT-(TAMRA)	(Thomas et al. 2007)
Sma-F	*S. marcescens*	16S rRNA	GGTGAGCTTAATACGTTCATCAATTG	(Iwaya A et al. 2005)
Sma-R	*S. marcescens*	16S rRNA	GCAGTTCCCAGGTTGAGCC	(Iwaya A et al. 2005)
Sma-probe	*S. marcescens*	16S rRNA	(6FAM)-TGCGCTTTACGCCCAGTAATTCCGA-(TAMRA)	(Iwaya A et al. 2005)
Calb-F	*C. albicans*	ITS2	GGGTTTGCTTGAAAGACGGTA	(Guiver et al. 2001)
Calb-R	*C. albicans*	ITS2	TTGAAGATATACGTGGTGGACGTTA	(Guiver et al. 2001)
Calb-probe	*C. albicans*	ITS2	(6FAM)-ACCTAAGCCATTGTCAAAGCGATCCCG-(TAMRA)	(Guiver et al. 2001)
Ec-F	*E. coli*	16S rRNA	TCCTACGGGAGGCAGCAGT	(Nadkarni et al. 2002)
Ec-R	*E. coli*	16S rRNA	GGACTACCAGGGTATCTAATCCTGTT	(Nadkarni et al. 2002)
Ec-probe	*E. coli*	16S rRNA	(6FAM)-GGAGTAAAGTTAATACCTTTGCTCATT-(TAMRA)	(Nadkarni et al. 2002)

Text S1

Title: Effects of Oral Moisturizing Gels Containing Propolis Following Head and Neck Radiotherapy: A Randomized Controlled Pilot Trial

Authors: Ryoma NAKAO[1]* and Takao UENO[2]

1. Department of Bacteriology I, National Institute of Infectious Diseases, Tokyo 162-8640, Japan.
2. Department of Dentistry, National Cancer Center Hospital, Tokyo 104-0045, Japan

* Corresponding author:

Ryoma Nakao, DDS, PhD
Department of Bacteriology I, National Institute of Infectious Diseases,
1-23-1 Toyama, Shinjuku-ku, Tokyo 162-8640, Japan.
Tel: +81-3-5285-1111, Fax: +81-3-5285-1163
Email: ryoma73@nih.go.jp

Running head: Propolis for radiotherapy-related oral complications

Supplementary text for "Materials and methods" section.

Patient selection

Patient selection for the trial was performed according to the following inclusion criteria: outpatients who had undergone radiotherapy with a dose of at least 50 Gy to the head and neck area, not less than 20 years old, and relatively good performance status ranging from a score of 0 to 2 according to the Eastern Cooperative Oncology Group (ECOG) [1]. Those who had received antimicrobial treatment in the three months before the study; or were smokers; edentulous in both jaws, or allergic to the components used in the treatments were excluded.

Preparation of gels with and without natural products or antimicrobial biocide

Propolis used in this study was collected in a region of Minas Gerais State, Brazil during the rainy season from October to May. *Baccharis dracunculifolia* was the botanical origin of this Brazilian propolis, which is classified as type-12 propolis [2]. Crude propolis was pulverized with a homogenizer and extracted in ethanol. After stirring at room temperature for 12 hours, the extract was evaporated until the solid content reached 55% (Yamada Bee Company, Inc., Okayama Japan), which was then standardized to contain a minimum of 8.0% artepillin C. The propolis concentrate was diluted with ethanol to yield 25 mg/mL (w/v, dry weight) of ethanol-extracted propolis. The curry leaf used in the present study was collected in a region of North Central Province, Sri Lanka (S&B Foods Inc., Tokyo, Japan). Turmeric used in the present study was collected in India (House Foods Inc., Tokyo, Japan). Ethanol-extracted curry leaf or the turmeric was obtained as a supernatant after stirring 1 g of curry leaf powder or 1 g of turmeric powder (House Foods Inc., Tokyo, Japan) into 20 mL of ethanol using a magnetic stirrer for 2 hours. Ethanol-extracted curry leaf was evaporated until the solid content reached 45 mg/mL (w/v, dry weight) to yield ethanol-extracted curry leaf , while ethanol-extracted turmeric was evaporated until the solid content reached 16 mg/mL (w/v, dry weight) to yield ethanol-extracted turmeric. The placebo gel was prepared by mixing 1 mL of food grade ethanol and 100 g of oral moisturizing gel with a homogenizing mixer. A low concentration of the food grade ethanol (1%) was added

to the placebo gel instead of an ethanol-extracted natural product without expectation of any antimicrobial activity, for standardizing all the gel contents except for the individual natural product extracts. Propolis 250 μg/mL (w/v) gel was prepared using the same method as for the placebo gel, except 1 mL of 25 mg/mL (w/v) ethanol-extracted propolis was added instead of food-grade ethanol. Curry leaf 450 μg/mL (w/v) gel was prepared using the same method as that for the placebo gel, except 1 mL of 45 mg/mL (w/v) of ethanol-extracted curry leaf was added instead of food-grade ethanol. Turmeric 160 μg/mL (w/v) gel was prepared using the same method as that for the placebo gel, except 1 mL of 16 mg/mL (w/v) of ethanol-extracted turmeric was added instead of food-grade ethanol. CHX 0.05% (w/v) gel was prepared by mixing with 1 mL of 5 % (w/v) CHX (Nichi-Iko Pharmaceutical Co., Ltd., Toyama, Japan) instead of 1 mL of food-grade ethanol in placebo gel. All five kinds of gels were packaged in 50-mL laminated tubes (Kansai Tube Co., Ltd., Osaka, Japan) and sealed by heating the open end, then stored at 4 °C before use.

Evaluation of clinical parameters

Symptoms of dryness and pain in the oral cavity were evaluated using a 100-mm visual analogue scale (VAS). We determined the degree of mouth dryness using a Mucus® moisture checker (Yoshida Co., Tokyo, Japan), according to the manufacturer's instructions. Oral hygiene status, oral dryness, gingivitis, and oral candidiasis were clinical parameters judged based on visual inspection by a single, trained dentist who was blinded to the type of treatment received by the subject.

Evaluation of microbiological parameters

Saliva was obtained and 1 mL samples were pretreated with Sputazyme®, an enzymatic reagent, to decrease the sample viscosity, according to the manufacturer's instructions (Kyokuto Co., Tokyo, Japan). Saliva samples were centrifuged for 5 minutes at 17,400 x *g* to obtain a pellet containing bacterial cells, with the pellets used as starting materials for bacterial DNA extraction. Total bacterial DNA was extracted from saliva samples using a ChargeSwitch gDNA Mini Bacteria kit (Invitrogen, Carlsbad, CA, USA), according to the manufacturer's instructions. For the last step of the purification,

purified DNA was eluted with 200 µL of elution buffer. The purity and yield of the DNA were determined by NanoDrop analysis (ND-1000, NanoDrop Technologies Montchanin, DE, USA). Purified DNA was subjected to a TaqMan-based quantitative real-time polymerase chain reaction (qPCR) assay to quantify the numbers of the following six major periodontopathic bacteria; *P. gingivalis* [3], *Tannerella forsythia* [4], *Treponema denticola* [5], *F. nucleatum* [6], and *Aggregatibacter actinomycetemcomitans* [3] , as well as four opportunistic pathogens; methicillin-resistant *Staphylococcus aureus* (MRSA) [7], *Escherichia coli* [8, 9], *Serratia marcescens* [10], and *Candida albicans* [11]. The number of total bacteria was quantified as the universal 16S rRNA gene copy number [9]. Real-time qPCR was performed using Premix Ex Taq (Probe qPCR) (Takara Bio, Shiga, Japan) with universal or species-specific primers and probes (Table S1), which were purchased from Eurofins Genomics (Tokyo, Japan) or Sigma-Aldrich Japan (Tokyo, Japan). Real-time PCR was performed in a 20-µL final volume in a reaction tube or plate using an ABI PRISM 7500 sequence detection system (Applied Biosystems, Foster, CA, USA). Two µL of DNA was added to 18 µL of premix solution containing 1x Premix Ex Taq, 0.4 µM of each primer, and 0.2 µM of a fluorogenic probe. The PCR conditions consisted of 1 cycle of 30 seconds at 95°C, followed by 40 cycles of 5 seconds at 95°C and 34 seconds at 60°C. The number of target copies in the reaction was deduced from threshold cycle (CT) values by referring to data from standard plasmids named pRN124, 125, 126, 130, and 133, which were constructed from a pTAKN-2 vector (2739 base pairs, kanamycin resistant, BioDynamics Laboratory Inc., Tokyo, Japan). pRN124 is comprised of a conserved sequence region of the 16S rRNA gene (467 nucleotides [nt]) from *P. gingivalis* strain ATCC 33277 (accession number: NC_002950.2) and was used as the standard plasmid for DNA-based quantification of total bacteria. pRN125 is comprised of a *P. gingivalis*-specific sequence region of the 16S rRNA gene (126 nt) from the ATCC 33277 strain (NC_002950.2), an *A. actinomycetemcomitans* -specific sequence region of the *hlyA* gene (147 nt) from the D11S-1 strain (NC_013416), and an *F. nucleatum*-specific sequence region of the 23S rRNA gene (101 nt) from the ATCC 25586 strain (NC_003454). pRN125 was used as a standard plasmid for DNA-based quantification of those three periodontopathic bacteria. pRN126 is comprised of a *T. forsythia*-specific sequence region of the 16S rRNA gene (162 nt) from the ATCC 43037 strain (NC_016610), a *T. denticola*-specific sequence region of the 16S rRNA gene (122 nt)

from the ATCC 35405 strain (NC_002967). pRN126 was used as a standard plasmid for DNA-based quantification of those two periodontopathic bacteria. pRN130 is comprised of a MRSA-specific sequence region of the *mecA* gene (88 nt), a *S. marcescens*-specific sequence region of the 16S rRNA gene (179 nt) from the DSM 30121 strain (AJ233431), and a *C. albicans*-specific sequence region of the internal transcribed spacer 2 (ITS2) gene (179 nt) from the DSM 30121 strain (AJ233431). pRN130 was used as a standard plasmid for DNA-based quantification of these three opportunistic pathogens. pRN133 is comprised of an *E. coli*-specific sequence region of the 16S rRNA gene (467 nt) from the MG1655 strain. pRN133 was used as a standard plasmid for DNA-based quantification of *E. coli*. The corresponding copy numbers were calculated based on the sizes and concentrations of the plasmid DNA.

Statistical analysis

Statistical analyses were performed for the microbiological and clinical variables according to group (placebo, CHX, curry leaf, propolis, turmeric), or timeline (baseline, final follow-up). One-way analysis of variance (ANOVA) followed by the Dunnett's multiple comparison test was used to statistically evaluate variables in each group. Using the mean values of the respective measurements before and after treatment, changes over time were calculated and tested with a Mann-Whitney U test. All values are expressed as the mean ± SD. P-values of 0.05 or less were considered to be statistically significant.

References

1. Oken MM, Creech RH, Tormey DC, et al.: Toxicity and response criteria of the Eastern Cooperative Oncology Group. *Am J Clin Oncol.* 1982;5(6):649-655.

2. Park YKI, M.; Alencar, S.M.; Moura, F.F.: Evaluation of Brazilian propolis by both physicochemical methods and biological activity. *Honey Bee Sci.* 2000;21:85-90.

3. Yoshida A, Suzuki N, Nakano Y, et al.: Development of a 5' fluorogenic nuclease-based real-time PCR assay for quantitative detection of Actinobacillus actinomycetemcomitans and Porphyromonas gingivalis. *J Clin Microbiol.* 2003;41(2):863-866.

4. Tada A, Takeuchi H, Shimizu H, et al.: Quantification of periodontopathic bacteria in saliva using the invader assay. *Jpn J Infect Dis.* 2012;65(5):415-423.

5. Kuboniwa M, Amano A, Kimura KR, et al.: Quantitative detection of periodontal pathogens using real-time polymerase chain reaction with TaqMan probes. *Oral Microbiol Immunol.* 2004;19(3):168-176.

6. Suzuki N, Yoshida A, Saito T, et al.: Quantitative microbiological study of subgingival plaque by real-time PCR shows correlation between levels of Tannerella forsythensis and Fusobacterium spp. *J Clin Microbiol.* 2004;42(5):2255-2257.

7. Thomas LC, Gidding HF, Ginn AN, et al.: Development of a real-time Staphylococcus aureus and MRSA (SAM-) PCR for routine blood culture. *J Microbiol Methods.* 2007;68(2):296-302.

8. Hansen WL, Beuving J, Bruggeman CA, et al.: Molecular probes for diagnosis of clinically relevant bacterial infections in blood cultures. *J Clin Microbiol.* 2010;48(12):4432-4438.

9. Nadkarni MA, Martin FE, Jacques NA, et al.: Determination of bacterial load by real-time PCR using a broad-range (universal) probe and primers set. *Microbiology.* 2002;148(Pt 1):257-266.

10. Iwaya A, Nakagawa S, Iwakura N, et al.: Rapid and quantitative detection of blood Serratia marcescens by a real-time PCR assay: its clinical application and

evaluation in a mouse infection model. *FEMS Microbiol Lett.* 2005;248(2):163-170.

11. Guiver M, Levi K, Oppenheim BA: Rapid identification of candida species by TaqMan PCR. *J Clin Pathol.* 2001;54(5):362-366.

Text S2

Title: Effects of Oral Moisturizing Gel Containing Propolis Following Head and Neck

Radiotherapy: Randomized Controlled Pilot Trial

Authors: Ryoma NAKAO[1]* and Takao UENO[2]

1. Department of Bacteriology I, National Institute of Infectious Diseases, Tokyo 162-8640, Japan.
2. Department of Dentistry, National Cancer Center Hospital, Tokyo 104-0045, Japan

* Corresponding author:

Ryoma Nakao, DDS, PhD
Department of Bacteriology I, National Institute of Infectious Diseases,
1-23-1 Toyama, Shinjuku-ku, Tokyo 162-8640, Japan.
Phone: +81-3-5285-1111, Fax: +81-3-5285-1163
E-mail: ryoma73@nih.go.jp

Running head: Propolis for radiotherapy-related oral complications

Legends for supplementary figures

Fig. S1

Improvements in oral hygiene (visual inspection), gingivitis (visual inspection), oral dryness (visual inspection), and oral dryness (Mucus®) were compared among the groups. Oral hygiene status was evaluated by visual inspection, according to the following criteria: no plaque accumulation as "Good" (Grade I); small amounts of plaque accumulation in oral cavity as "Moderate" (Grade II); overall plaque accumulation in oral cavity as "Poor" (Grade III). Gingivitis status was evaluated by visual inspection, according to the following criteria: no inflammation as "Good" (Grade I); mild redness or swelling as "Moderate" (Grade II); strong redness, severe swelling, or pus discharge as "Poor" (Grade III). Oral dryness status was evaluated by visual inspection, according to the following criteria: no symptom of dryness as "Good" (Grade I); foamy saliva as "Moderate" (Grade II); increased viscosity of saliva as "Poor" (Grade III); severe oral dryness of the whole mucosal surface as "Very poor" (Grade IV). Oral dryness status was also evaluated by Mucus®. All values are expressed as the mean ± SD.

Fig. S2

Total bacteria in saliva. Copy numbers of universal 16S rRNA in saliva samples collected from the subjects were quantified by real-time PCR, and shown for each before (black bars) and after (grey bars) intervention. The subjects were divided into the placebo (n = 6), CHX (n = 5), curry leaf (n = 4), propolis (n = 5), and turmeric (n = 6) groups. The distribution of the total bacterial numbers in all subjects (n = 25) is also shown. Horizontal axes in all figures represent copy numbers of universal 16S rRNA ($\log_{10}$).
